# Percutaneous closure of perimembranous ventricular septal defect using patent ductus arteriosus occluders

**DOI:** 10.1371/journal.pone.0206535

**Published:** 2018-11-15

**Authors:** Hieu Lan Nguyen, Quang Tan Phan, Dung Duc Doan, Linh Huynh Dinh, Hieu Ba Tran, Saima Sharmin, Julian Johny Thottian, Hoyoun Won, Wang Soo Lee, Seung Yong Shin, Truong Quang Nguyen, Sang Wook Kim

**Affiliations:** 1 Intervention Center, Vietnam National Heart Institute, Ha Noi, Vietnam; 2 Intervention Center, Hanoi Medical University Hospital, Hanoi, Vietnam; 3 Intervention Center, Quang Nam Central General Hospital, Quang Nam, Vietnam; 4 Intervention Center, Chung-Ang University Hospital, Seoul, South Korea; 5 Intervention Center, Westfort Group Hospital, Kerala, India; University of Milano, ITALY

## Abstract

**Objectives:**

To assess the safety and efficacy of percutaneous closure of perimembranous ventricular septal defect (PmVSD) using patent ductus arteriosus (PDA) occluders.

**Background:**

Widespread use of conventional PmVSD closure devices has been limited by unacceptable high rate of complete heart block (CHB). The elegant design of PDA occluders is supposed to ease implantation, increase closure rate and minimize damage to adjacent structures. Thus, PDA occluders may reduce complications, especially the CHB, and offer a good alternative for PmVSD closure.

**Method:**

From September 2008 to October 2015, patients who underwent attempted percutaneous VSD closure using PDA occluders were included in the study. Patient demographics, echocardiography measurements, procedure details and follow-up data until October 2017 were collected.

**Results:**

In total, 321 patients with a mean age of 15.5±12.6 years and mean a weight of 33.3±20.5 kg were included in this study. The mean defect size was 4.8±2.1 mm. Implantation was successful in 307 (95.6%) patients. The median follow-up time was 63 months (24 to 108 months). The closure rates were 89.5%, 91.5%, and 99.3% after the procedure 24 hours, 6 months and 2 years, respectively. Major complications occurred in 5 (1.7%) patients during the procedure and follow-up, including persistent CHB in 2 (0.7%) patients and device embolization in 3 (1.0%) patients. No death, disability, or other major complication was detected.

**Conclusion:**

Percutaneous closure of PmVSD using PDA occluders is feasible, safe and efficacious in selected patients.

## Introduction

Ventricular septal defect (VSD) is among the most common congenital heart diseases in both children and adults [1]. Although surgical closure has been accepted as the treatment of choice, it is associated with surgical complications, long hospital stays, sternotomy, skin scar and psychosocial effect. Since the first percutaneous closure of VSD performed by Lock in 1988 [2], along with the development of many closure devices and deployment techniques, percutaneous PmVSD closure was also commonly followed in many institutes world-wide [3–10]. However, widespread use of these conventional PmVSD closure devices has been limited recently by the unacceptable high rate of CHB compared with surgical repair [11–14]. Therefore, the search for alternative devices with good performance and low complication rate, especially CHB, is crucial in clinical practice.

PDA occluders are single disc devices based on nitinol wire meshes and originally designed for percutaneous PDA closure. They combine the ease of implantation with a high occlusion rate and a low rate of complication [15, 16]. Owning to the specific design for PDA closure, these devices may also be suitable for other defects with a duct shape and similar pathological characteristics of left-to-right shunt, especially for PmVSD [17, 18]. Compared with conventional PmVSD occluders, such as Amplazer Membranous VSD Occluder, the PDA occluders are smaller, softer and more flexible devices that can be released, retrieved, or repositioned easily through a smaller delivery system. In addition, the single disc design of these devices removes the clamp force and reduces radial stress to the ventricular septum. These advantages are supposed to reduce or even eliminate the drawbacks of conventional PmVSD occluders and make PDA occluders good alternative devices for percutaneous PmVSD closure.

## Methods

### Patient selection

**Fig 1:**
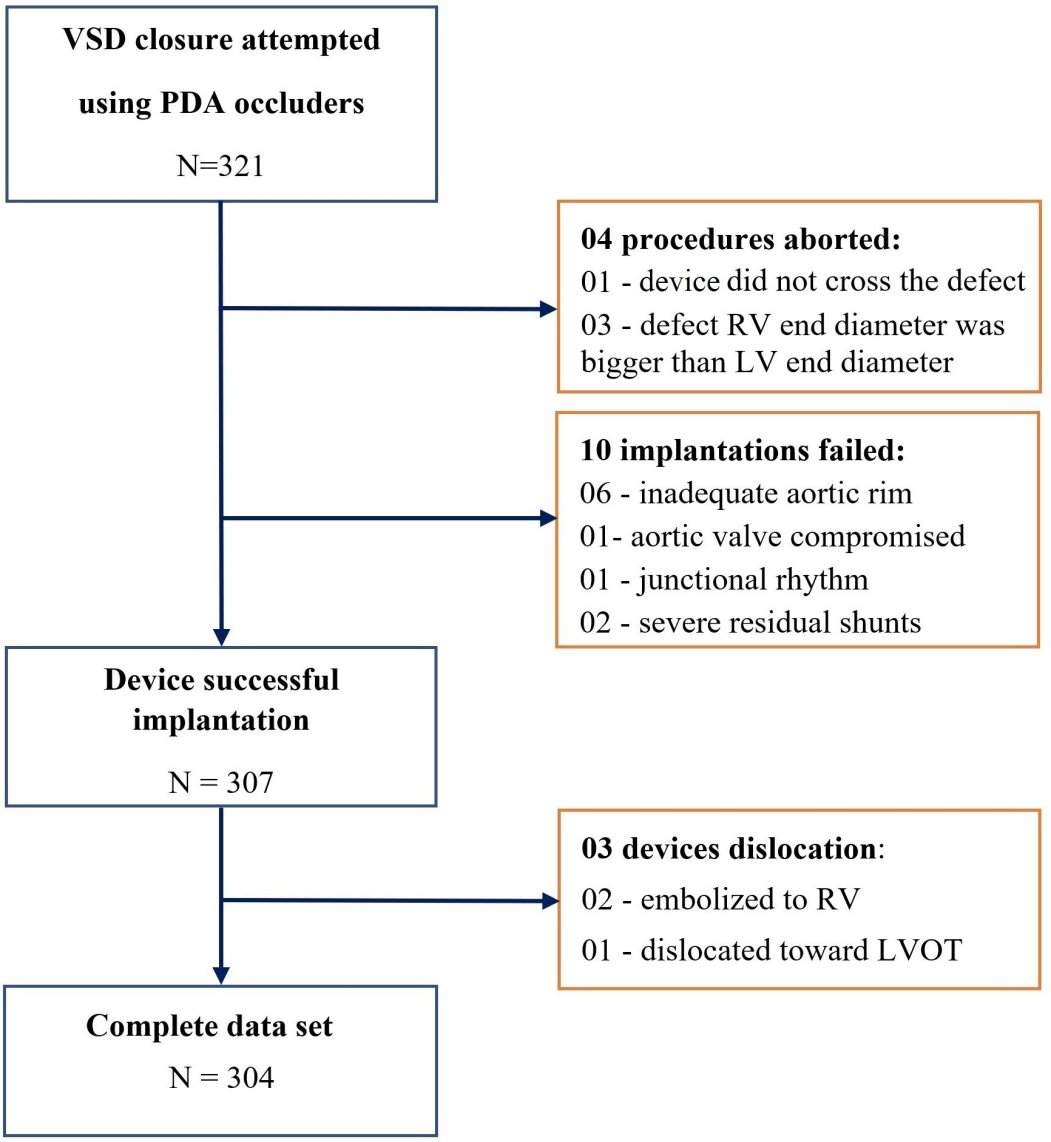
Study flow chart.

From September 2008 to October 2015, 321 patients (including 130 patients under 6 years of age), who underwent attempted percutaneous closure of VSD using PDA occluders at Vietnam National Heart Institute (n = 221), Ha Noi Medical University Hospital (n = 60), Ha Noi Heart Institute (n = 25) and Quang Nam Central General Hospital (n = 15) were retrospectively included in this study (**S1 File**). Inclusion criteria for percutaneous PmVSD closure using PDA occluders were left to right shunt, Qp/Qs > 1.5, age > 5 months, weight > 5 kg, and at least one of the following: dilation of the left ventricle or left atrium on two-dimensional echocardiography, symptoms of heart failure, failure to thrive or cardiomegaly on chest radiography. Patients with concomitant lesions requiring cardiac surgery, pulmonary vascular resistance >8 Wood units, significant aortic regurgitation, aortic valve prolapse and right-to-left shunt were excluded from device closure. The study flow chart is presented in **Fig 1**.

This study was approved by the Institutional Review Board of Quang Nam Central General Hospital and was carried out in accordance with the Declaration of Helsinki. As this study was a retrospective medical record-based study and all study subjects were de-identified, the need for written consent was waived.

### Devices

**Fig 2:**
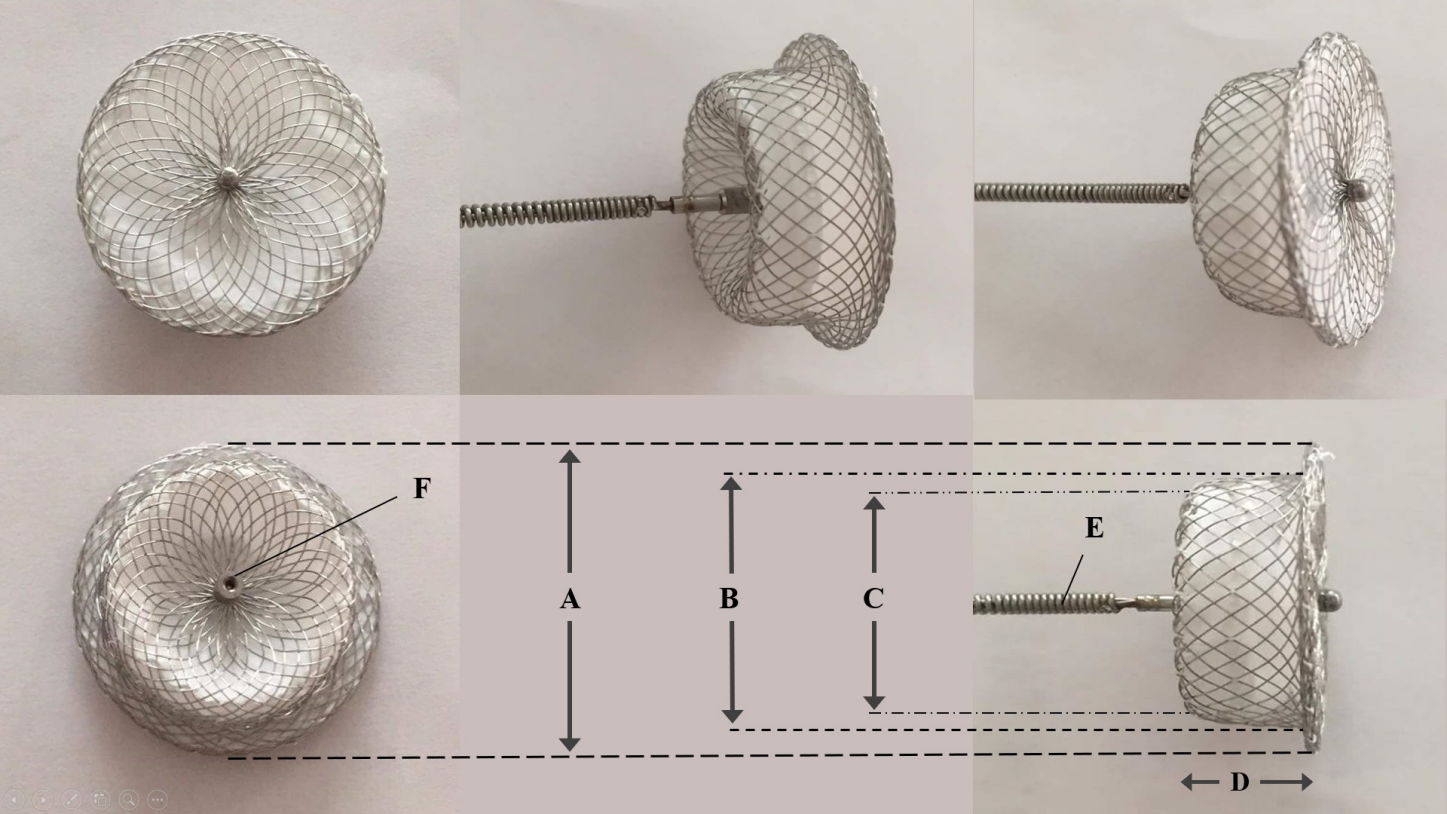
Typical design of a PDA occluder. (A) Left ventricular retention disc diameter. (B) Left ventricular side diameter. (C) Right ventricular side diameter. (D) Device length. (E) Delivery cable. (F) Internal threat, where the cable connects to the device.

The three types of PDA occluders used in this study were Amplatzer Duct Occluder (St. Jude Medical Inc., United States), Cocoon Duct Occluder (Vascular Innovations Co. Ltd., Thailand), and Cera PDA Occluder (Lifetech Scientific Co. Ltd., China). These devices have similar self-expanding and self-centering nitinol wire mesh design with a small single retention disk and a cylindrical main body (**Fig 2**). A polyester fabric is sewn inside the device with polyester thread, inducing thrombosis that closes the communication. The right ventricular (RV) end of the device will fit in the RV side of the PmVSD while the bigger left ventricular (LV) end and retention disc provides secure positioning in the LV ampulla of the defect. The Amplatzer Duct Occluder has available sizes of 6/4, 8/6, 10/8, 12/10, 14/12 and 16/14 mm, where the first number refers to the LV end diameter and the second number indicates the RV end diameter of the device. The biggest size of the Cocoon Duct Occluder is 20/18 mm and the Cera PDA Occluder size is up to 24/22 mm. The selected device size (RV end diameter) was 2 mm bigger than the smallest PmVSD diameter measured on echocardiography and left ventriculogram. The delivery system includes a delivery catheter (ranges from 5 to 8 French), dilator, delivery cable, loading device, hemostasis valve and plastic vise.

### Procedure

**Fig 3:**
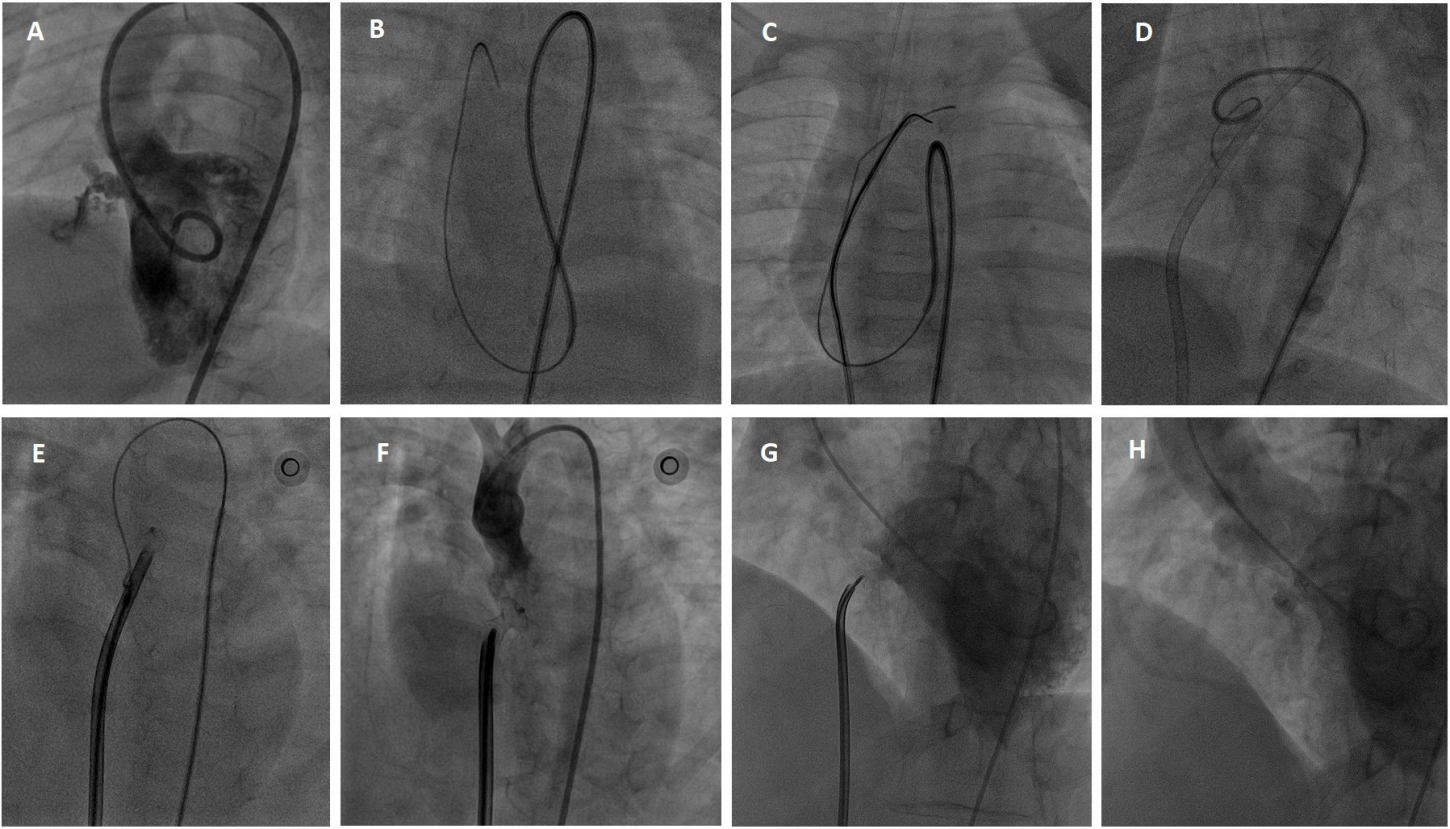
Percutaneous PmVSD closure procedure. (A) Left ventriculogram. (B) The guidewire crosses from the left ventricle to the right ventricle through the defect and then to the superior vena cava. (C) The guidewire is snared at the superior vena cava to make an arterio-venous loop. (D) The delivery catheter is introduced from venous access through the loop to the ascending aorta. (E) The device is loaded in the delivery catheter and partly released in the aorta. (F) Check aorta gram before device release. (G) Check left ventriculogram before device release. (H) The final left ventriculogram after device release shows the device is in good position and there is no residual shunt.

An informed written consent was obtained from all patients or their guardians before intervention. The procedure was performed under general anesthesia in all infants and small children or local anesthesia in the older patients. Access was obtained through the right femoral vein and right femoral artery. Heparin 100 UI/kg and prophylactic antibiotic were given intravenously before standard right and left cardiac catheterizations were performed. Left ventriculography at 55° left anterior oblique and 30° cranial projection was used to profile the defect (**Fig 3A**). Location, size of the VSD, and its relationship with the aortic valve were assessed. The VSD size (the smallest diameter of the defect, usually located on the RV side), VSD ampulla size (the largest diameter of the defect, usually located on the LV side) and the VSD length were measured.

Through the VSD, a 0.35-inch Terumo guidewire (Terumo Corporation, Japan) was introduced from the LV to RV, right atrial, superior vena cava, and was subsequently captured by a snare advancing from the femoral vein to make an arteriovenous loop (**Fig 3B and 3C**). Through this loop, the delivery catheter was introduced from the venous access to the RV, LV, and to the ascending aorta (**Fig 3D)**. The closure device was connected to the cable, advanced to the defect through the delivery catheter, and then deployed under fluoroscopic and echocardiographic guidance (**Fig 3E–3H**). Left ventriculogram, aortogram, and echocardiography were performed before release to confirm proper device position.

### Follow-up

Continuous ECG monitoring was used during the first 24 hours after the procedure to detect arrhythmia. Echocardiography was performed immediately and 24 hours after the procedure to check for complications and residual shunt. Urine analysis was indicated within 24 to 48 hours after the procedure to detect hemolysis. Aspirin 5 mg/kg per day was administered for 6 months in all patients. The follow-up echocardiography, ECG and clinical examination were scheduled at 1month, 6 months, and every 12 months after the procedure. The follow-up data was available until October 2017.

### Data collection and definitions

The data collected included patient demographic information, clinical presentations, echocardiography measurements, procedure details and complications occurred during procedure and follow-up. Echocardiographic measurements consisted of defect size, LV ejection fraction (EF), LV diastolic diameter (LVDd), left atrial (LA) diameter, pulmonary systolic pressure, sub aortic rim length and the systolic gradient through the defect. Procedural details recorded included procedure time, fluoroscopy time, pulmonary pressure, Qp/Qs ratio, the defect size on left ventriculogram, and the type and size of devices used. Additionally, the reasons for aborted procedures or failed implantations were also documented.

Heart failure was diagnosed using Framingham criteria. Failure to thrive in children was defined by weight less than the 5^th^ percentile for age and sex. On echocardiography, LA enlargement was defined as a LA to aortic ratio > 1.5 in the long-axis parasternal view. LV enlargement was defined as LVDd ≥ 2 standard deviation for the body surface area. A residual shunt was considered to be present if Color-Doppler echocardiography showed a left to right jet across the VSD. It was classified as small (jet ≤1 mm), moderate (jet 2–4 mm), or large (jet ≥4 mm).

Major complication was defined as procedure-related death, life-threatening adverse events, or those required surgery, such as device embolism, myocardial perforation, vessel rupture, severe residual shunt, severe hemolysis, valvular injury or persistent complete heart block that required permanent pacemaker insertion. Complications categorized as minor were non-fatal complications and regressed spontaneously or with medication such as access site hematoma, hemolysis that only required medication, blood transfusion because of blood loss, transient complete heart block, bundle branch block, fascicular block, first degree or Mobitz I type AV block, rash, fever, etc. Total complication was the sum of minor and major complications.

Procedure success was defined by the absence of major complications and appropriate position of the implanted device on echocardiography 24 hours after the procedure.

### Statistical analysis

Statistical analyses were performed with SPSS Statistics 23.0 (IBM Corp., Armonk, NY, USA). Continuous variables are expressed as mean ± standard deviations or median and quartiles. Categoric data are presented as frequencies and percentages.

## Results

From September 2008 to October 2015, 321 patients (43.6% male) underwent attempted percutaneous closure of PmVSD using PDA occluders. The baseline characteristics of participants are presented in **Table 1**. The mean age was 15.5±12.6 years (from 7 months to 58 years, patients with age < 6 years accounted for 40.5%). The mean weight was 32.4±20.4 kg (from 5.8 to 75.0 kg, patients with weight < 10 kg accounted for 12.2%). Of these patients, 22.7% had symptoms of heart failure, 27.1% had recurrent respiratory infection, 32.4% failed to thrive, and only 18.7% were asymptomatic. On echocardiography, the mean VSD size was 4.3±1.3 mm, the mean pulmonary artery systolic pressure was 35.3±7.8 mmHg, mean sub aortic rim was 3.4±1.4 mm and 156 (48.6%) patients had PmVSD with aneurysm. Cardiomegaly on chest radiograph was detected in 74 (23.1%) patients. One patient who had concomitant ASD and two patients who had PDA were percutaneously closed during the index procedure.

**Table 1:** Baseline characteristics.

Participants	N = 321
Age (year)	15.0±12.6
Age groups, n (%)	
≤6 years	130 (40.5%)
6–16 years	58 (18.1%)
>16 years	133 (41.4%)
Male, n (%)	140 (43.6%)
Weight (kg)	32.4±20.4
Clinical presentations	
Heart failure, n (%)	73 (22.7%)
Recurrent pneumonia, n (%)	87 (27.1%)
Failure to thrive, n (%)	104 (32.4%)
Dyspnea, n (%)	90 (28.0%)
Palpitation, n (%)	11 (3.4%)
Chest pain, n (%)	64 (19.9%)
Asymptomatic, n (%)	60 (18.7%)
Echocardiography	
VSD size (mm)	4.3±1.3
VSD ampulla size (mm)	7.0±2.5
Systolic gradient* (mmHg)	89.0±18.8
Sub aortic rim (mm)	3.4±1.4
PAPs (mmHg)	35.3±7.8
EF (%)	66.4±6.2
LVDd (mm)	43.2±8.5
LVDs (mm)	27.2±5.9
Aneurysm pmVSD, n (%)	156 (48.6%)
Concomitant defect	
ASD, n	1
PDA, n	2
Cardiomegaly on chest radiograph	72 (22.4%)

VSD: ventricular septal defect; PAPs: pulmonary artery systolic pressure; EF: left ventricular ejection fraction; LVDd: left ventricular end diastolic dimension; LVDs: left ventricular end systolic dimension;

*: pressure gradient through the defect;

ASD: atrial septal defect; PDA: patent ductus arteriosus.

The procedure and device data are presented in **Table 2.** The mean procedure time was 45.8±16.9 minutes. The mean Qp/Qs ratio measured on catheterization was 2.1±0.6. On left ventriculography, mean VSD size was 4.8±2.1 mm (from 2.0 to 14.0 mm), mean VSD length was 5.9±1.6 mm (from 3.6 to 12.0 mm) and mean sub aortic rim was 3.5±1.8 mm (from 1.2 to 8.0 mm). Altogether, 97 Amplatzer Duct Occluders, 149 Cocoon Duct Occluders and 61 Cera PDA Occluders were implanted.

**Table 2:** Procedure and device data.

Variable	Value
Fluoroscopy time (minute)	13.9±4.2
Procedure time (minute)	45.8±16.9
Catheterization	
PA systolic pressure (mmHg)	34.2±12.4
PA diastolic pressure (mmHg)	16.5±6.2
PA mean pressure (mmHg)	21.5±7.8
Qp/Qs ratio	2.1±0.6
Left ventriculogram	
VSD size (mm)	4.8±2.1
VSD ampulla size (mm)	7.8±3.0
VSD length (mm)	5.9±1.6
Sub aortic rim (mm)	3.5±1.8
Device type	
Amplatzer Duct Occluder, n (%)	97 (31.6%)
Cera PDA Occluder, n (%)	61 (19.9%)
Cocoon Duct Occluder, n (%)	149 (48.5%)
Device size	
RV end (mm)	8.0±3.2
LV end (mm)	10.1±3.2
Devices selected	
6x4 mm, n (%)	43 (14.1%)
8x6 mm, n (%)	91 (29.9%)
10x8 mm, n (%)	75 (24.7%)
12x10 mm, n (%)	49 (16.1%)
14x12 mm, n (%)	19 (6.3%)
16x14 mm, n (%)	12 (3.9%)
18x16 mm, n (%)	13 (4.3%)
20x18 mm, n (%)	02 (0.7%)

PA: pulmonary artery; Qp: pulmonary blood flow; Qs: systemic blood flow; LV: left ventricle, RV: right ventricle.

The procedure success and closure rates are presented in **Table 3**. Of the 321 patients included in this study, 307 (95.6%) patients had successful implantation with the PDA occluders. Immediate closure of the defect without any residual shunt after deployment was achieved in 236 (77.6%) patients. Further echocardiographic follow-up showed that the closure rate gradually increased up to 89.5%, 91.5%, 97.4% and 99.3% at 24 hours, 6months, 1 year, and 2 years after the procedure, respectively.

**Table 3:** Procedural success and closure rate.

Variable	Value
Procedure attempted, n	321
Procedure success, n (%)	307/321 (95.6%)
Reasons for procedure failure, n (%)	14/321 (4.6%)
Inadequate aortic rim	06
Aortic valve compromised	01
Junctional rhythm	01
Severe residual shunt	02
Device couldn’t pass the defect	01
Unsuitable defect anatomy *	03
Closure rate on echocardiography	
Immediate closure, n (%)	236 (77.6%)
After 24 hours, n (%)	272 (89.5%)
After 6 months, n (%)	278 (91.5%)
After 1 year, n (%)	296 (97.4%)
After 2 years, n (%)	302 (99.3%)

RV: right ventricle; LV: left ventricle; LVOT: left ventricular outflow tract;

*: the diameter of the defect on the RV side was larger than that on the LV side.

The adverse events during the procedure and follow-up are presented in **Table 4**. The median follow-up time was 63 months (24–108 months). Most of the adverse events were categorized as minor complication (11.7%). Major complications occurred in 5 (1.7%) patients. Device embolization was detected in 3 (1.0%) patients. In the first case, a 16 months old boy, the device dislocated to the LV out flow tract 3 days after the procedure. In the two other cases, a 3-year-old girl and a 16-year-old boy, the devices embolized to the RV 36 hours and 6 weeks after deployment, respectively. These patients underwent cardiac surgery for device removal and VSD closure with good recovery. Persistent CHB that required permanent pacemaker insertion occurred in 2 (0.7%) patients, including a 6-year-old boy at 15 days and a 40-year-old female at 5 months after the procedure. There was no death, disability or other major complication detected.

**Table 4:** Adverse events.

Events	N = 307
Total complications, n (%)	41 (13.4%)
Major complications, n (%)	05 (1.7%)
Persistent complete heart block, n (%)	02 (0.7%)
Device embolization, n (%)	03 (1.0%)
Minor complications, n (%)	36 (11.7%)
Access site hematoma, n (%)	05 (1.6%)
Hemolysis diminished with medication, n (%)	06 (2.0%)
Blood transfusion because of blood loss, n (%)	02 (0.7%)
New trivial aortic regurgitation, n (%)	03 (1.0%)
Transient complete heart block, n (%)	02 (0.7%)
Junctional rhythm, n (%)	01 (0.3%)
Left bundle branch block, n (%)	02 (0.7%)
Left fascicular block, n (%)	01 (0.3%)
Right bundle branch block, n (%)	01 (0.3%)
Second degree AV II (Mobitz I), n (%)	01 (0.3%)
First degree AV I block, n (%)	06 (2.0%)
Paroxysmal AF, n (%)	01 (0.3%)
Other (rash, fever >38.5°C), n (%)	05 (1.6%)

PM: pace maker; AV: atrio—ventricular; AF: atrial fibrillation

## Discussion

Percutaneous closure of VSD using the conventional PmVSD occluders, either symmetrical or asymmetrical double disc design, has largely been abandoned because of unacceptable high CHB rate documented in the literature [13, 14, 19–21]. Early CHB may be the consequence of significant direct mechanical trauma caused by the delivery system or device deployment during the procedure, while late CHB may be due to fibrosis, compression, or inflammation of the conduction system [15, 20, 21]. The conventional PmVSD occluders have a high tendency to cause damage to the ventricular septum and other adjacent structures because of the big delivery system needed for large profile devices, along with the high clamping force caused by double disc design and high radial stress due to high device stiffness [22]. Thus, another device with lower profile and ease of implantation may reduce the trauma, clamp force, and radial stress to the ventricular septum and therefore, decrease the CHB complication rate [22, 23]. Among available devices, PDA occluders may have these characteristics and may offer a promising alternative to conventional VSD occluders with better outcome and fewer complications.

Previous studies showed that infants and small children had lower procedure success and higher major complication rates of percutaneous PmVSD closure [3, 20, 24]. The same results were observed in this study, where most of the participants were children and the percentage of patients aged less than 6 years was also high, up to 40.5% (**Table 5**). For percutaneous VSD closure in these small children, the PDA occluders with a small delivery system (6–8 French), the ease of implantation and, experienced operators are crucial keys to increase procedural success rate and minimize the complications.

**Table 5:** Comparisons of the feasibility, efficacy and safety among age groups.

Age group	≤6 years (1) n = 130	6–16 years (2) n = 58	>16 years (3) n = 133	p (1) vs. (2)	p (1) vs. (3)	p (2) vs. (3)
**Procedure success** (%)	90.8	98.3	99.2	0.061	**0.002**	0.554
**Closure rate**						
Immediate closure (%)	80.2	82.1	73.5	0.758	0.214	0.203
After 24 hours (%)	92.2	91.1	86.4	0.793	0.138	0.138
After 6 months (%)	93.1	92.9	89.4	0.953	0.305	0.305
After 1 year (%)	96.6	96.5	98.5	0.967	0.323	0.323
After 2 years (%)	100.0	99.4	99.6	0.149	0.384	0.348
**Complication**						
Total complication (%)	19.5	8.8	9.8	0.081	**0.030**	0.817
Minor complication (%)	17.2	7.1	9.1	0.073	0.056	0.781
Major complication (%)	2.5	1.8	0.8	0.744	0.262	0.539

Careful defect sizing and proper device selection are also important to reduce the complications of VSD closure [25, 26]. While undersized devices increase the rate of device embolization and residual shunt, oversized devices may cause more damage to the adjacent structures and result in complications such as CHB, aortic or tricuspid valvular injury, etc. The device size selected in this study, which was only 2 mm bigger than the defect size, helped to maintain necessary radial force to prevent device embolization and to minimize the radial stress on the septal wall [26, 27].

Another important factor that greatly impacted the success of the procedure was the sub aortic rim length. A short aortic rim makes the implanted device less stable, and the retention disc may compromise and cause damage to the aortic valve. For cases with a small aortic rim and no aneurysmal tissue, we might choose a smaller device, accepted mild to moderate residual shunt to minimize the damage to aortic valve. However, respecting that surgical VSD closure is currently the treatment of choice and patient safety is the priority, in cases with unstable device position (6 cases), severe residual shunt (2 cases) or aortic valve compromised (1 case) before release, we stopped the implantation and recommended cardiac surgery for the patients.

The defect shape also has an important influence during the PmVSD closure procedure. In this study, 40.5% of patients had PmVSD with aneurysms. For these cases, the conventional double disc device size must be large enough to cover the whole aneurysm and may cause further complications [28]. However, implantation of PDA occluders will be more convenient in PmVSD with aneurysm because the retention disc can be put entirely within the aneurysm and the cylindrical portion of the device secures in an opening of the aneurysm on the RV side [15, 24, 27]. Therefore, the device will not contact the aortic valve and create minimum pressure on the ventricular septum. On the contrary, in patients with the defect RV end diameter much bigger than the LV end diameter (03 cases), there was no way the cylindrical PDA occluders with smaller RV end diameter could achieve a stable position inside the defect. So, we abandoned the procedure and sent the patients for surgical closure.

In this study, the procedure success (95.6%) and the immediate closure rate (77.6%) were high and comparable to those of the other studies, using the conventional double disc devices (such as Amplatzer Membranous VSD Occluder) [3–5, 24, 29], later off-label use devices (such as Amplatzer Duct Occluder, Amplatzer Duct Occluder II) [15, 30, 31], new specific VSD closure devices (such as Nit-Occlud Lê VSD Coil) [22, 23] or modified devices (such as modified VSD Occluder in China) [32] for PmVSD closure. Furthermore, the closure rate gradually increased up to 99.3% after 2 years, the pulmonary systolic pressure on echocardiography decreased and became steady after the procedure 6 months (**S1 Fig**), along with the improvement of clinical presentations observed in most patients during the follow-up showed good efficacy of the PDA occluders for PmVSD closure.

Device embolization occurred in 3 patients during the hospital stay and follow-up. In the first patient, a 16-month-old baby, the device (Cera PDA Occluder) dislocated to the LV out flow tract 3 days after the procedure. In two other patients, a 3-year-old girl and a 16-year-old boy, the devices (both Cocoon Duct Occluder) embolized to the RV 36 hours and 6 weeks after deployment, respectively. These patients then underwent cardiac surgery for device removal and VSD closure with good recovery. Noticeably, all device embolization occurred in the first years of this study. The reasons might be errors in defect size estimation that lead to under-sized device selection or the problems in the device deployment technique that lead to suboptimal device positioning, which occurred during the early phase of the study. Furthermore, these cases had high risks for device embolization, such as defect size > 5 mm, aortic rim < 2 mm and moderate residual shunt existed before device release. Therefore, careful patient selection, gaining operators experience and improving closure techniques are important to reduce device embolization in percutaneous PmVSD closure with PDA occluders.

The huge cornerstone that makes the widespread use of percutaneous VSD closure with devices has been limited is the unacceptable high rate of CHB in the literature [11, 20, 24, 33, 34]. This complication may happen very early or very late after the procedure [24, 35, 36], may be reversible with medication, or may become persistent and require permanent pacemaker insertion [37,38]. Because of this reason, percutaneous VSD closure has been largely abandoned in many countries and surgical closure has been accepted as the contemporary standard therapy for PmVSD. In our study, persistent CHB complication happened in 2 patients. The first patient was a-6-year-old boy, who was diagnosed with CHB on ECG 15 days after PmVSD closure with a 10×8 mm Amplazer Duct Occluder. The second patient was a 40-year-old female, who had symptoms of CHB 5 months after PmVSD closure with a 12×10 mm Cocoon Duct Occluder. These patients were treated with permanent pacemakers and there was no sign of sinus rhythm recovery during the follow-up. Thus, PmVSD closure using the PDA occluders does not completely free all patients from CHB. However, the CHB rate in our study was lower than that in most previous studies regarding percutaneous VSD closure (**S2 Fig**). In comparison with percutaneous VSD closure using conventional devices, the incidence of CHB after surgical VSD closure in the literature was lower, ranging from 0% to 2.2% [39–49], and a mean of 1.1% according to meta-analysis [50]. The incidence of CHB in our study (0.7%) was comparable with that of surgical closure (**S2 Fig)**. In other words, percutaneous PmVSD closure using duct occluders may be a good alternative therapy to surgical closure in suitable patients.

### Limitations and strengths

This was a retrospective study with a limited number of participants and a wide age range. Besides, the devices were personally selected by the operators based on the preference, availability, and familiarity with these devices. Furthermore, every procedure was performed with at least one operator that had copious experience in VSD closure; therefore, the outcomes of the procedures in this study might not represent routine practice. Even though this was a retrospective study, the strict protocol of percutaneous PmVSD closure in each institute before, during and after the procedure made the collected data comprehensive and accurate.

## Conclusion

Percutaneous closure of PmVSD using PDA occluders is feasible, safe and efficacious in selected patients. More improvement of device design, operators experience, and implantation techniques along with careful patient selection may further minimize the drawbacks and make percutaneous PmVSD closure with suitable devices become an acceptable alternative to cardiac surgery in the future.

## Supporting information

S1 File The raw data of all participants included in the study. (XLSX)

S1 Fig Echocardiographic follow-up data. (TIF)

S2 Fig Incidence of complete heart block after VSD closure. (TIF)
