# Supplementary figures and images for: Percutaneous closure of perimembranous ventricular septal defect using patent ductus arteriosus occluders
Source: PLoS One. 2018 Nov 15;13(11):e0206535. doi: 10.1371/journal.pone.0206535 (PMC6237304; doi:10.1371/journal.pone.0206535)

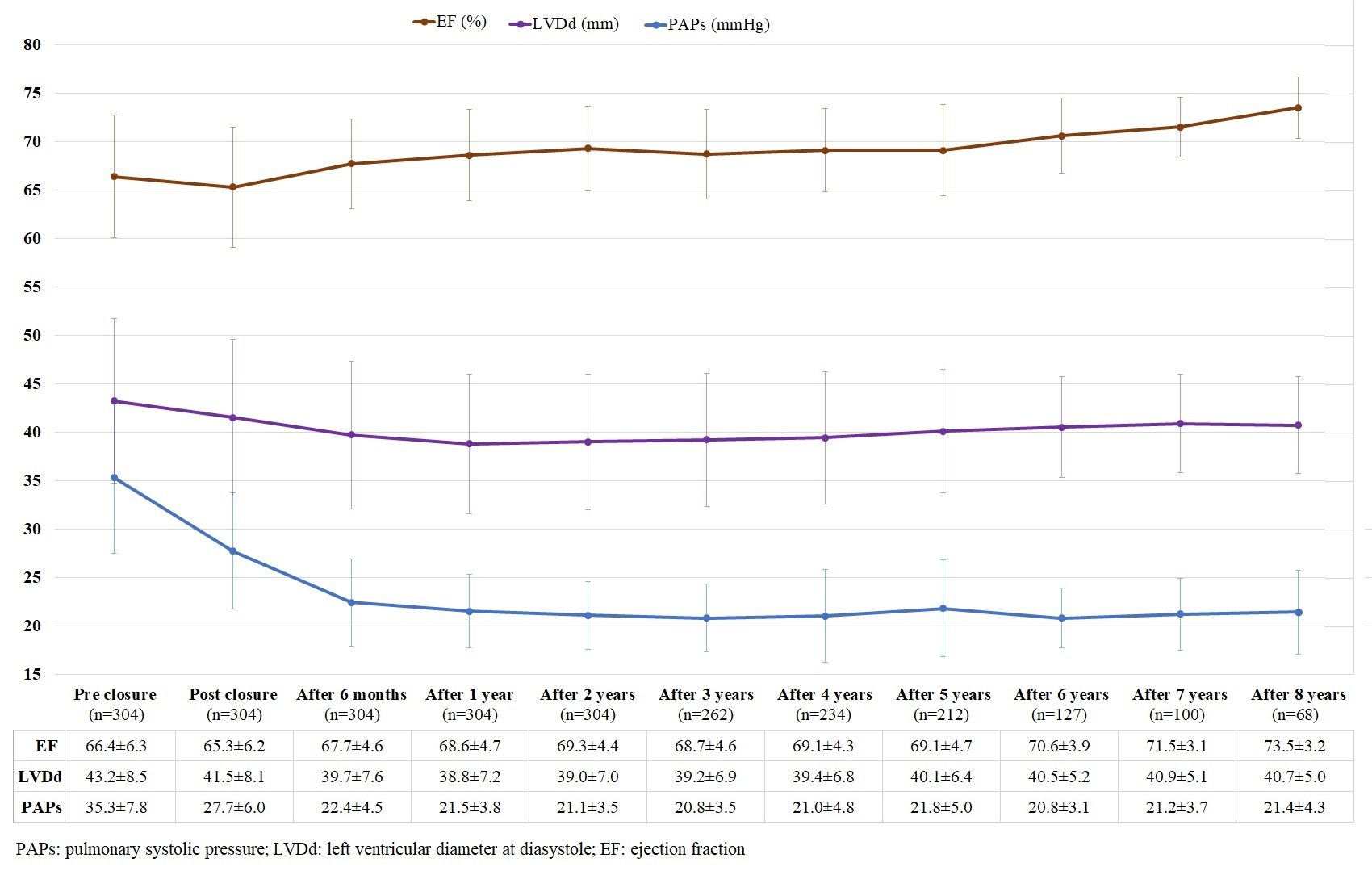

Supplement: S1 Fig — (TIF) [file pone.0206535.s002.tif]

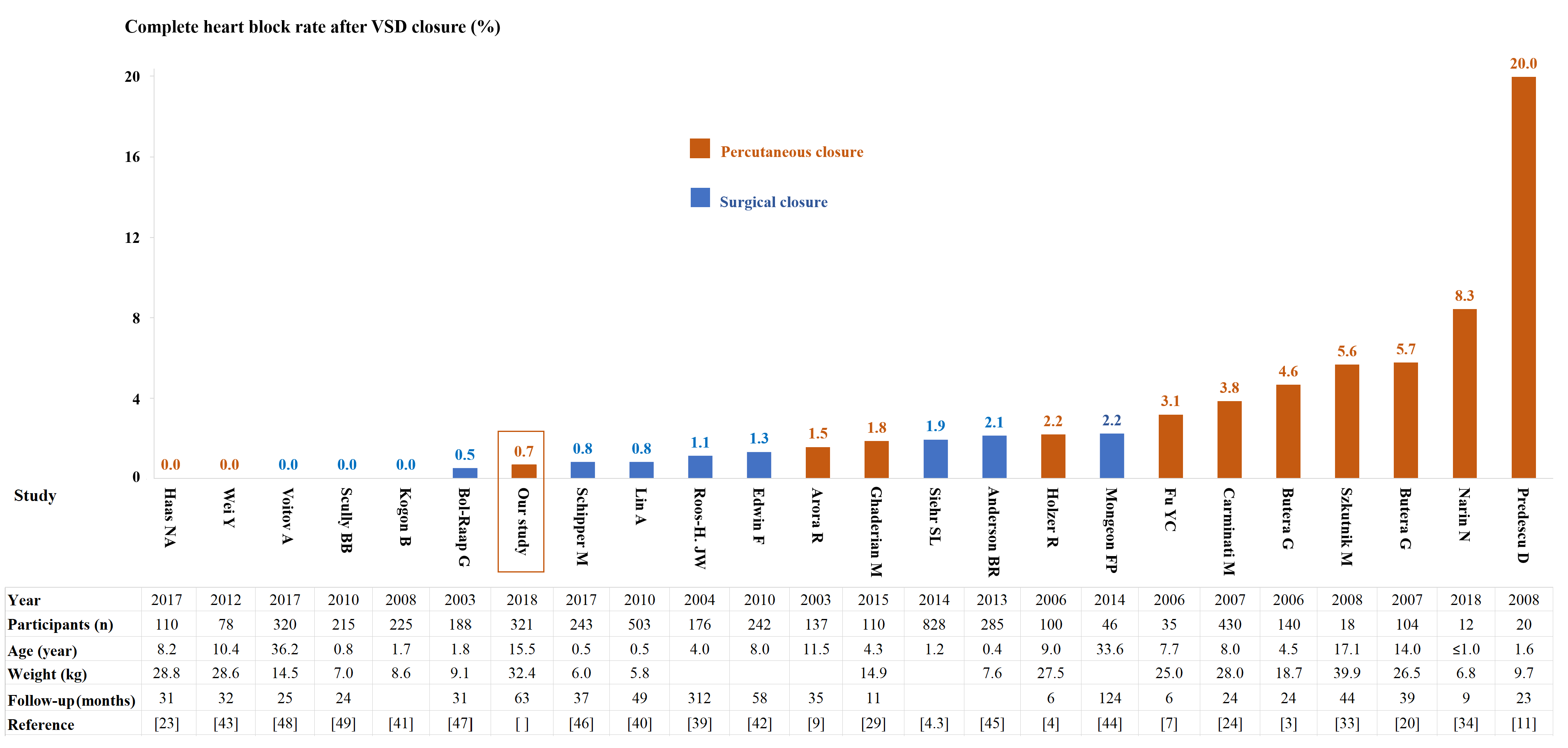

Supplement: S2 Fig — (TIF) [file pone.0206535.s003.tif]
